# Modulation of magnetoencephalography alpha band activity by radiofrequency electromagnetic field depicted in sensor and source space

**DOI:** 10.1038/s41598-021-02560-0

**Published:** 2021-12-03

**Authors:** Jasmina Wallace, Lydia Yahia-Cherif, Christophe Gitton, Laurent Hugueville, Jean-Didier Lemaréchal, Brahim Selmaoui

**Affiliations:** 1 Department of Experimental Toxicology and Modeling (TEAM), Institut National de l’Environnement Industriel et des Risques (INERIS), Parc Technologique Alata, BP 2, 60550 Verneuil-en-Halatte, France; 2 PériTox Laboratory, UMR-I 01 INERIS, Université de Picardie Jules Verne, 80025 Amiens, France; 3 Centre De NeuroImagerie De Recherche (CENIR), Institut du Cerveau et de la Moelle épinière (ICM), 75013 Paris, France; 4 Inserm U 1127, CNRS UMR 7225, Institut du Cerveau et de la Moelle épinière (ICM), Sorbonne Université, 75013 Paris, France

**Keywords:** Neurophysiology, Neuroscience, Physiology

## Abstract

Several studies reported changes in spontaneous electroencephalogram alpha band activity related to radiofrequency electromagnetic fields, but findings showed both an increase and a decrease of its spectral power or no effect. Here, we studied the alpha band modulation after 900 MHz mobile phone radiofrequency exposure and localized cortical regions involved in these changes, via a magnetoencephalography (MEG) protocol with healthy volunteers in a double-blind, randomized, counterbalanced crossover design. MEG was recorded during eyes open and eyes closed resting-state before and after radiofrequency exposure. Potential confounding factors, known to affect alpha band activity, were assessed as control parameters to limit bias. Entire alpha band, lower and upper alpha sub-bands MEG power spectral densities were estimated in sensor and source space. Biochemistry assays for salivary biomarkers of stress (cortisol, chromogranin-A, alpha amylase), heart rate variability analysis and high-performance liquid chromatography for salivary caffeine concentration were realized. Results in sensor and source space showed a significant modulation of MEG alpha band activity after the radiofrequency exposure, with different involved cortical regions in relation to the eyes condition, probably because of different attention level with open or closed eyes. None of the control parameters reported a statistically significant difference between experimental sessions.

## Introduction

The extensive use of the mobile phone (MP) has necessarily increased the exposure of the population to radiofrequency electromagnetic fields (RF-EMF). The biological effects of RF-EMF and their consequences on human health have received much attention from scientists.

In particular, several studies investigated the effect of RF-EMF on the human brain. Some studies looked at the effect on cerebral blood flow^1–3^ or on the cerebral hemodynamic response via functional-MRI^4,5^. Other studies looked at electrical brain activity via electroencephalography (EEG) and one of the most consistent non-thermal RF-EMF effects experimentally observed so far on the EEG resting-state was the modulation of the alpha band spectral power^6,7^. Indeed, the bulk of these investigations (about 80%) showed EEG spectral power changes related to RF-EMF exposure^6^, whereas the others did not report any effects^8–13^. In general, studies on adolescents^11,14^ or elderly people^14^ did not show any effects on the cortical activity of the waking EEG at rest related to Global System for Mobile Communications (GSM) MP exposure compared to young adult volunteers^14–17^. Even though some studies found a significant modification of the spectral power of alpha band and other frequency bands (delta, theta, beta and gamma) related to MP exposure, the majority showed that the EEG resting-state modification was mostly found on alpha band^6^. However, all together these studies showed heterogeneous findings about the alpha band modulation, since some of them reported an increase of the spectral power of the alpha band^14,15,18–23^ while others reported a decrease^16,17,24–27^. An explanation for these heterogeneous findings could lie in discrepancies between studies concerning the use of methodologies and experimental protocols, frequencies and intensities applied by exposure systems and criteria for participant inclusion^6,7^. Findings in this research field could differ between studies also because of different statistical analysis^7^, and it has been suggested to apply multiple comparisons and effect size statistical approaches to check the rate of false positive results and the potential inflation of type I errors, as well as the sufficient sample size to detect a subtle effect^28^. Finally, results regarding RF-EMF non-thermal effects on human brain cortical activity at rest and alpha band are still inconclusive. It is noteworthy that also several physiological and environmental factors can modify EEG power. These factors include hormone level variations (cortisol^29^, melatonin^30^, ovarian-menstrual cycle hormones^31^), emotional stress behavior^32^ and consumption of caffeine^33^, alcohol, nicotine and illicit drugs^34^. Rigorous and well performed studies should control these potential cofounding factors to limit bias when analyzing power spectral density modulation related to RF-EMF exposure^6^.

In general, when compared to the underlying mechanisms of the thermal RF-EMF biological effects, the non-thermal ones are far from being fully understood and need further investigations. The influence of water polarization on hydrogen bonding forces between water molecules under microwaves exposure has been proposed to have a non-thermal impact on neurotransmitters transit time and neuron resting potential^35,36^. Similarly, some in vitro^37^ and in vivo^38,39^ studies showed modifications in the neuronal rates of spontaneous activity and in neurotransmission processes related to RF-EMF exposure^40,41^. Other studies showed also cortical myelin sheath damage^42^ and decreased expression of calcium channels in hippocampus^43,44^ as consequence of RF-EMF exposure. Recently, a thermal mechanism explaining MP exposure effects (with 1 W/kg and 2 W/kg of SAR), has also been suggested showing that a modulation of the human EEG activity was accompanied by thermoregulatory changes^45^.

As there are heterogeneous results about RF-EMF exposure effect on the EEG alpha band spectral power, we deemed interesting to further characterize its modulation by RF-EMF, by using the magnetoencephalography (MEG) combined with an individual anatomical cerebral MRI which could be an alternative to the limited spatial resolution of EEG applied in this context. Indeed, as reviewed recently^6,7^ few studies considered more than 21 electrodes in the study of the resting-state electrical brain activity and RF-EMF exposure. Moreover, a previous study researched RF-EMF GSM signal effects (with an exposure at 1800 MHz) on MEG brain activity of healthy subjects during encoding-retrieval tasks, recording MEG with 148 magnetometers whole-head system^46^. Findings revealed changes in the early task-specific components of the event-related fields during the exposure to RF-EMF^46^. So, to the best of our knowledge, a MEG protocol analysis in sensor and source space using a whole-head MEG system with 102 MEG sensor triplets has not been applied before in order to investigate the potential impact of RF-EMF on spontaneous alpha band activity at rest.

It is worth mentioning that, as in EEG, MEG records cortical phenomena with millisecond temporal resolution, but its main advantage is a higher spatial accuracy in both surface recording and source reconstruction. The topographic spatial profile of MEG data computed in the sensor space is visually and quantitatively less blurred and warped by changes of electrical conductivity between brain, skull and scalp compared with EEG signals originating from the same physiological brain source^47^.

For these reasons, the aim of our study was to replicate our previous results on EEG resting-state and MP^17^ investigating the effect of the MP exposure on the waking spontaneous alpha band MEG activity in sensor and source space of healthy young adults to highlight cortical areas involved in these changes.

Since RF-EMF emissions with lower frequency (and longer wavelength) are characterized by greater penetration depth into the body than higher ones with a more superficial specific absorption rate (SAR) distribution^48^, we focused our study on the effects of the exposure to second generation (2G) GSM MP technology at 900 MHz. Indeed, the most GSM signal applied worldwide includes the GSM-900 frequency band, which works at 890–915 MHz in uplink and 935–960 MHz in downlink with a frequency pulse-modulated signal, emitting 577 µs pulses every 4.6 ms repeated at a rate of 217 Hz, as well as the DCS-1800 frequency band between 1710 and 1785 MHz in uplink and 1805–1880 MHz in downlink. The more recent MP technologies of third generation (3G) and fourth generation (4G) operate at different frequencies. More precisely, the 3G technology using the Wideband Code Division Multiple Access (WCDMA) standard RF signals, also called Universal Mobile Telecommunication System (UMTS), mainly operate in Europe and Asia at 1920–1980 MHz in uplink and at 2110– 2170 MHz in downlink. The 4G technology based on the Long-Term Evolution (LTE) service utilizes different frequency range bands in European countries, such as 1710–1785 MHz (uplink) and 1805–1880 MHz (downlink), as well as a 2500–2570 MHz (uplink) and 2620–2690 MHz (downlink). It is noteworthy that nowadays 2G GSM technology is still largely used worldwide^49^.

Furthermore, to limit bias related to potential confounding factors in our study, we firstly assessed heart rate and heart rate variability (HRV), as indices for autonomic nervous system activity^50^. Secondly, we used biochemistry assays to analyze salivary concentration of biomarkers of stress. Such salivary biomarkers include cortisol, an indicator for the hypothalamic–pituitary–adrenal axis (HPA)^51^, chromogranin A and alpha amylase, both indicators for the sympatho-adreno-medullary system^52,53^. Finally, we measured caffeine concentration in saliva by High-Performance Liquid Chromatography (HPLC).

## Results

### MEG sensor space results for entire alpha band (8–12 Hz)

MEG resting-state with eyes-open (EO) and eyes-closed (EC) was recorded before RF-EMF exposure (baseline phase from Run 1 to Run 2) and after RF-EMF exposure (post-exposure phase from Run 9 to Run 12) during two recording sessions in a crossover, randomized, double-blind and counterbalanced experimental design with healthy volunteers.

We first estimated MEG power spectral densities in sensor space for both magnetometers and gradiometers considering the entire alpha band in the rage of 8–12 Hz. Results of three-way repeated-measure ANOVA showed statistically significant differences for time factor (two levels: baseline, post-exposure), eyes condition factor (two levels: EO, EC) and exposure condition factor (two levels: real, sham). Considering time factor, significant differences of alpha power across MEG recordings were depicted by MEG magnetometers and gradiometers placed at frontal and parietal regions during Run 9 (one significant cluster involving fronto-parietal magnetometers with cluster F-statistic = 30.40, Monte Carlo *p* < 0.001, and one significant cluster involving fronto-parietal gradiometers with cluster F-statistic = 31.65, Monte Carlo *p* < 0.001). Eyes condition factor showed for all recording runs significant differences of alpha power analysis depicted by both sensor types widespread over the scalp (one significant cluster involving parietal, frontal, occipital and temporal magnetometers, with cluster F-statistic = 45.99, Monte Carlo *p* < 0.001, and gradiometers with cluster F-statistic = 46.38, Monte Carlo *p* < 0.001). The alpha band powers were significantly modulated from Run 9 to Run 11 at left temporo-parietal regions in exposure condition factor (one significant cluster at temporo-parietal magnetometers with cluster F-statistic = 8.66, Monte Carlo *p* < 0.01, and one significant cluster at temporo-parietal gradiometers with cluster F-statistic = 8.10, Monte Carlo *p* < 0.01).

**Figure 1:**
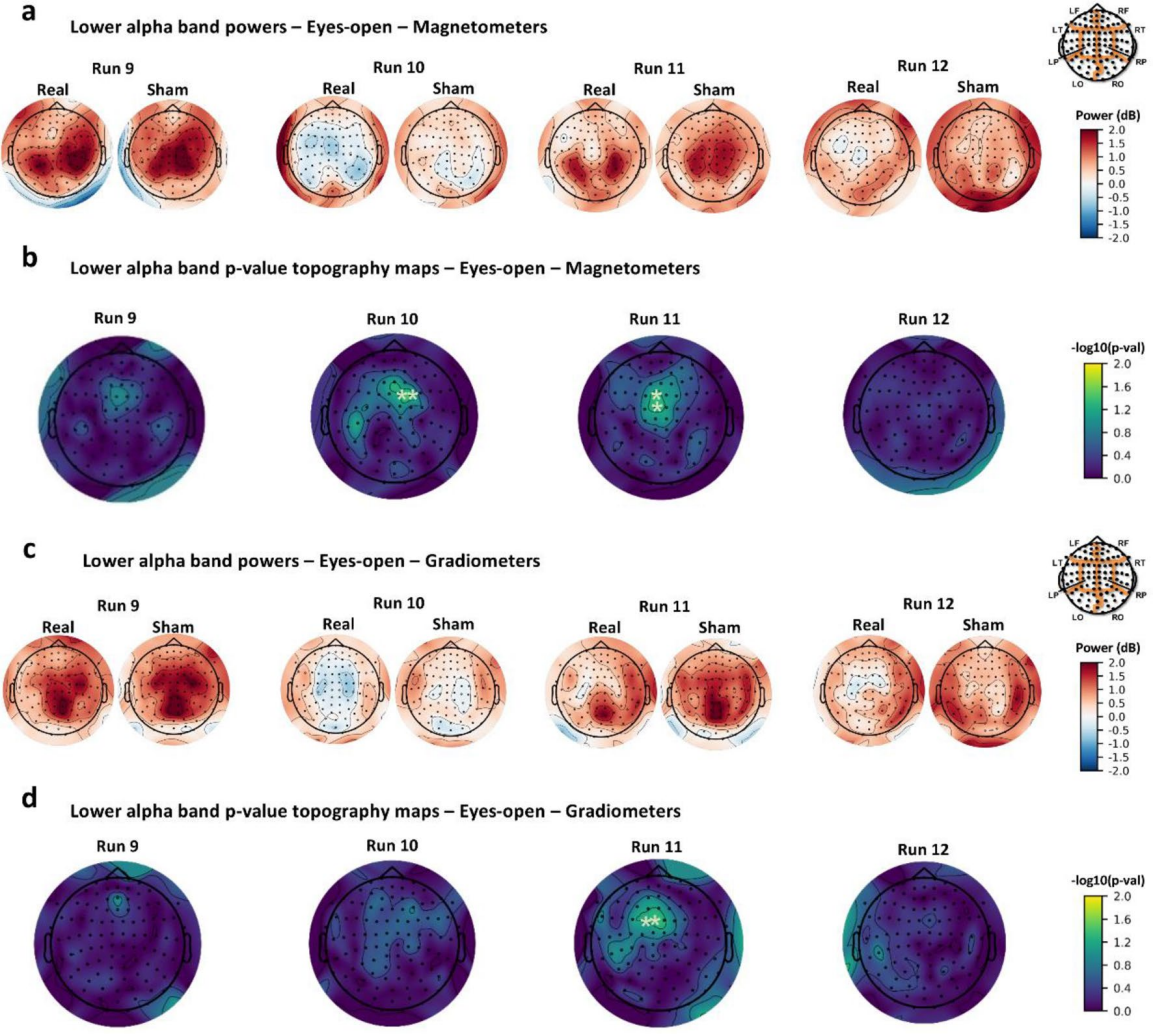
MEG sensor space results for the lower alpha band with magnetometers during eyes-open recordings (**a**, **b**) and gradiometers during eyes-open recordings (**c**, **d**). Power topography maps show baseline-corrected power values computed in sensor space for each sensor during runs of the real and the sham exposure session for 29 subjects and plotted according to the 102 MEG sensor triplets layout (LF, left frontal region; RF, right frontal region; LT, left temporal region; RT, right temporal region; LP, left parietal region; RP, right parietal region; LO, left occipital region; RO, right occipital region). *p* value topography maps show the results of one-way ANOVA on MEG baseline-corrected lower alpha band power of RF-EMF post-exposure sessions (real vs. sham) at sensor level analysis. *p* values were computed for each sensor for each run (*p < 0.05).

**Figure 2:**
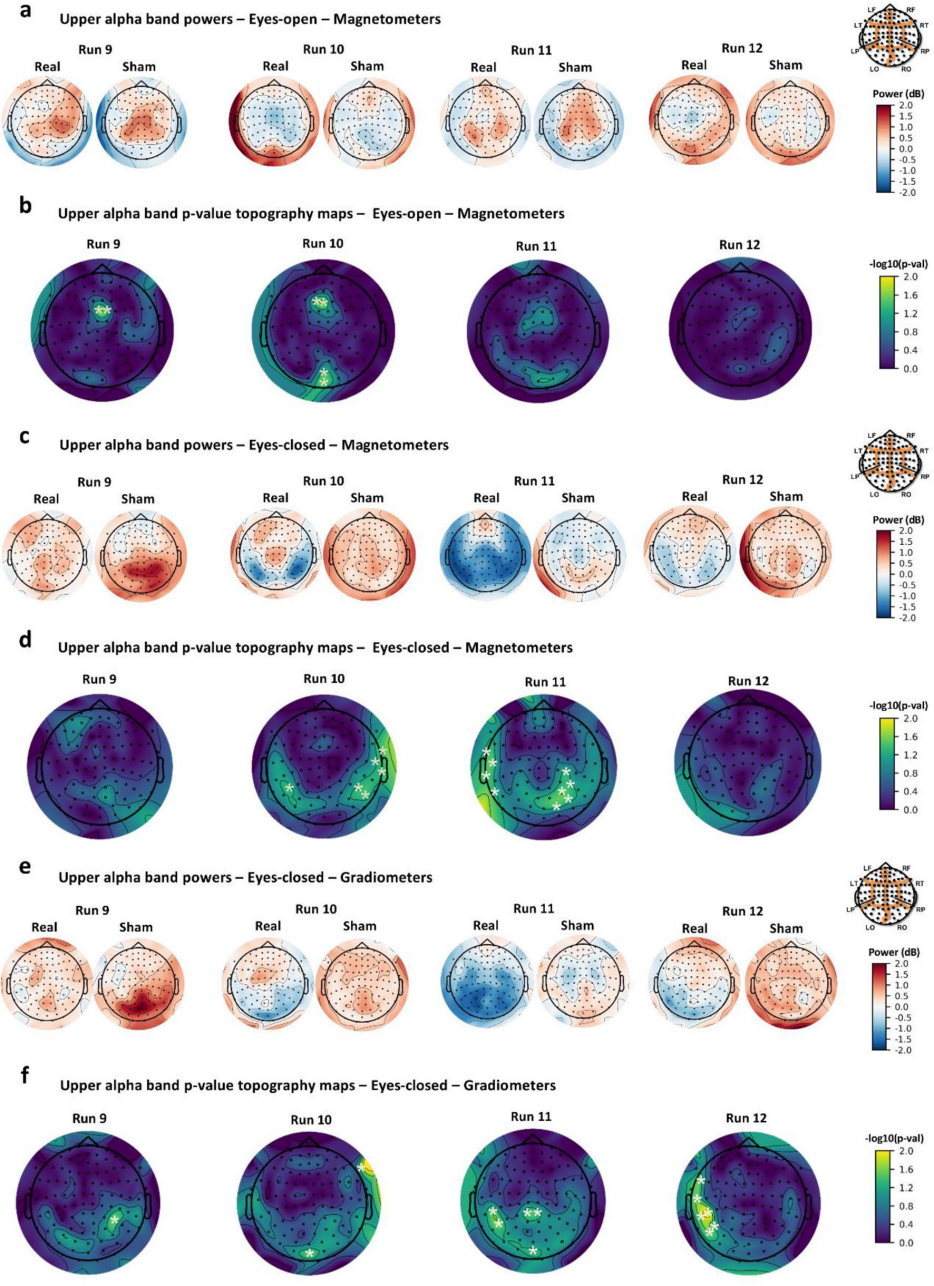
MEG sensor space results for the upper alpha band with magnetometers during eyes-open recordings (**a**, **b**), with magnetometers during eyes-closed recordings (**c**, **d**) and with gradiometers during eyes-closed recordings (**e**, **f**). Power topography maps show baseline-corrected power values computed in sensor space for each sensor during runs of the real and the sham exposure session for 29 subjects and plotted according to the 102 MEG sensor triplets layout (LF, left frontal region; RF, right frontal region; LT, left temporal region; RT, right temporal region; LP, left parietal region; RP, right parietal region; LO, left occipital region; RO, right occipital region). *p* value topography maps show the results of one-way ANOVA on MEG baseline-corrected upper alpha band power of RF-EMF post-exposure sessions (real vs. sham) at sensor level analysis. *p* values were computed for each sensor for each run (*p < 0.05).

To further characterize the RF-EMF exposure effect, real and sham RF-EMF post-exposure alpha band powers during EO and EC recordings from magnetometers and gradiometers were baseline-corrected and compared (one-way ANOVA). These results are shown in Figs. 1 and 2, respectively, of Supplementary Information and resumed in Table 1 of Supplementary Information. Considering the magnetometers power spectral density analysis, we found a statistically significant decrease of real post-exposure phase power relative to the sham, depicted by left fronto-parietal sensors during EO Run 9 (one significant cluster localized in fronto-parietal region with cluster F-statistic = 4.38 and Monte Carlo *p* < 0.05) and EO Run 10 (one significant cluster localized in fronto-parietal region with cluster F-statistic = 5.20 and Monte Carlo *p* < 0.05) and by right occipital sensors during EC Run 11 (one significant cluster at occipital region with cluster F-statistic = 3.54 and Monte Carlo *p* < 0.05). The results of gradiometers power spectral density analysis showed a statistically significant power decrease in the real post-exposure period relative to the sham depicted at right parietal location during EO Run 11 (one significant cluster detected at parietal region with cluster F-statistic = 4.24 and Monte Carlo *p* < 0.05) and at left temporo-occipitals gradiometers during EC Run 11 (one significant cluster at occipital region with cluster F-statistic = 4.38 and Monte Carlo *p* < 0.05) and Run 12 (one significant cluster at temporo-occipital region with cluster F-statistic = 5.19 and Monte Carlo *p* < 0.05).

### MEG sensor space results for lower and upper alpha sub-bands

Secondly, we estimated the MEG power spectral densities in sensor space of both magnetometers and gradiometers for lower and upper alpha sub-bands, in the range of 8–10 Hz and 10–12 Hz, respectively. Results of three-way repeated-measure ANOVA showed statistically significant differences of both lower and upper alpha powers for time factor (two levels: baseline, post-exposure), eyes condition factor (two levels: EO, EC) and exposure condition factor (two levels: real, sham). Considering time factor, significant differences of lower alpha power across MEG recordings were depicted by MEG magnetometer (one significant cluster at fronto-parietal region during Run 9 with cluster F-statistic = 38.66, Monte Carlo *p* < 0.001, and during Run 12 with cluster F-statistic = 14.61, Monte Carlo *p* < 0.01) and by gradiometers (one significant cluster at frontal region during Run 9 with cluster F-statistic = 49.75, Monte Carlo *p* < 0.001, and during Run 12 with cluster F-statistic = 16.86, Monte Carlo *p* < 0.01). Eyes condition factor showed for all recording runs significant differences of lower alpha power analysis depicted by both sensor types widespread over the scalp (one significant cluster involving parietal, frontal, occipital and temporal magnetometers with cluster F-statistic = 56.22, Monte Carlo *p* < 0.001, and gradiometers with cluster F-statistic = 56.67, Monte Carlo *p* < 0.001). Lower alpha band power was significantly modulated at left temporo-occipital regions in exposure condition factor as depicted by magnetometers from Run 9 to Run 11 (one significant cluster at temporo-occipital region with cluster F-statistic = 7.14, Monte Carlo *p* < 0.01) and by gradiometers from Run 9 to Run 11 (one significant cluster at temporo-occipital region with cluster F-statistic = 6.89, Monte Carlo *p* < 0.01).

On the other hand, considering time factor, significant differences of upper alpha power were depicted by MEG magnetometer after exposure during Run 9 (one significant cluster at fronto-parietal region with cluster F-statistic = 12.83, Monte Carlo *p* < 0.01) and by gradiometers during Run 9 (one significant cluster at fronto-parietal region with cluster F-statistic = 11.23, Monte Carlo *p* < 0.01). Eyes condition factor showed for all recording runs significant differences of upper alpha power analysis depicted by both sensor types widespread over the scalp (one significant cluster involving parietal, frontal, occipital and temporal magnetometers with cluster F-statistic = 35.33, Monte Carlo *p* < 0.001, and gradiometers with cluster F-statistic = 42.76, Monte Carlo *p* < 0.001). Finally, the upper alpha band power was significantly modulated at left temporo-occipital regions in the exposure condition factor across all runs as depicted by magnetometers (one significant cluster at temporo-occipital region with cluster F-statistic = 9.78, Monte Carlo *p* < 0.01) and by gradiometers (one significant cluster at temporo-occipital region with cluster F-statistic = 11.42, Monte Carlo *p* < 0.01).

Then, to further characterize the RF-EMF exposure effect, real and sham RF-EMF post-exposure powers of lower and upper alpha band during EO and EC recordings were baseline-corrected and compared (one-way ANOVA). Considering the EO recording blocks, the lower alpha baseline-corrected power decreased in the real post-exposure period relative to the sham with a statistically significant difference during Run 10, as detected by parietal magnetometers (one significant cluster localized in parietal and central sensors with cluster F-statistic = 4.70, Monte Carlo *p* < 0.05) and during Run 11 (a significant cluster in parietal sensors with cluster F-statistic = 4.30, Monte Carlo *p* < 0.05), as detected by parietal magnetometers (Fig. 1a,b) and gradiometers (Fig. 1c,d). Considering the baseline-corrected power of lower alpha band during EC recordings, the power spectral density clearly decreased in the real post-exposure relative to sham, especially at temporal-occipital magnetometers and gradiometers from Run 9 to Run 12. However, these findings did not show a statistically significant difference between two RF-EMF post-exposure conditions. These results are resumed in Table 1 of Supplementary Information.

Results showed that only parietal and occipital magnetometers revealed a statistically significant modulation of upper alpha baseline-corrected power during Run 9 and Run 10 with EO condition. In this case, during the real post-exposure phase compared to sham, the power spectral density modulation varied across the scalp areas, since it was significantly reduced at the central scalp region and slightly increased at the occipital one (with cluster F-statistic = 5.70, Monte Carlo *p* < 0.05), as shown in Fig. 2a,b. Otherwise, baseline-corrected power spectral density of upper alpha band clearly decreased in the real post-exposure phase relative to the sham during EC condition from Run 9 to Run 12, covering mainly the temporal-occipital area of the scalp (Fig. 2c). Magnetometers revealed this statistically significant modulation during Run 10 and 11 at the temporal-occipital area and also at the parietal area during Run 11, as shown in Fig. 2d (one significant cluster at occipital sensors, cluster F-statistic = 5.12, Monte Carlo *p* < 0.05). In addition, gradiometers revealed a statistically significant RF-EMF effect (power decrease) that occurred for the duration of the post-exposure recording phase, detected firstly at the parietal region and then also at the temporal and occipital sensors as shown in Fig. 2e,f (two significant clusters at occipital sensors in Run 11 and temporal sensors in Run12, cluster F-statistic = 8.01, Monte Carlo *p* < 0.01). These results are resumed in Table 1 of Supplementary Information.

### MEG source space results for lower and upper alpha sub-bands

**Figure 3:**
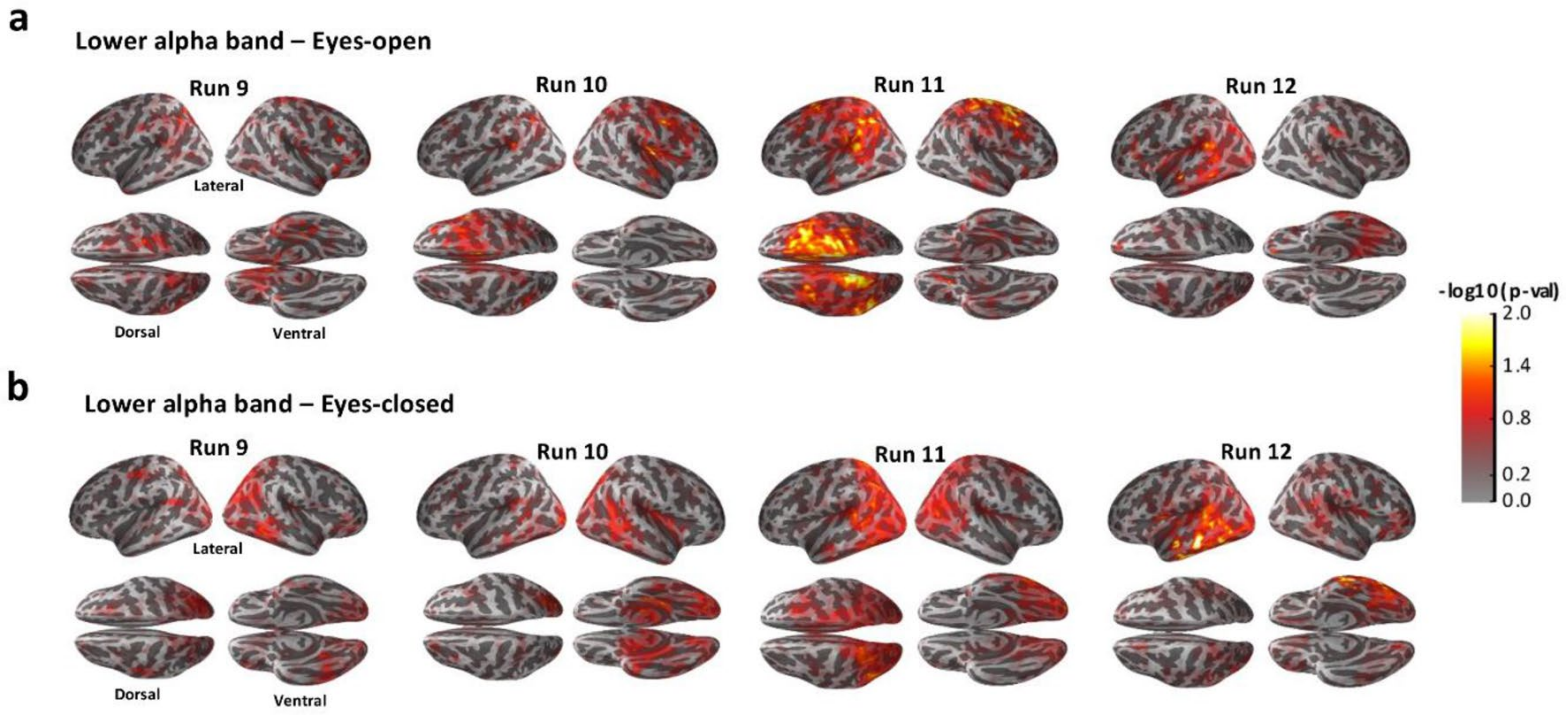
*p* value maps of one-way ANOVA on MEG baseline-corrected lower alpha band power spectral density of RF-EMF post-exposure sessions (real vs. sham) at source level analysis during eyes-open recordings (**a**) and eyes-closed recordings (**b**). *p* values were computed for each MEG source location (4098 current dipoles per hemisphere) for 29 subjects (*p*  < 0.05). The cortex is shown inflated with gyri darker than sulci, with lateral (left and right), dorsal and ventral visualizations.

**Figure 4:**
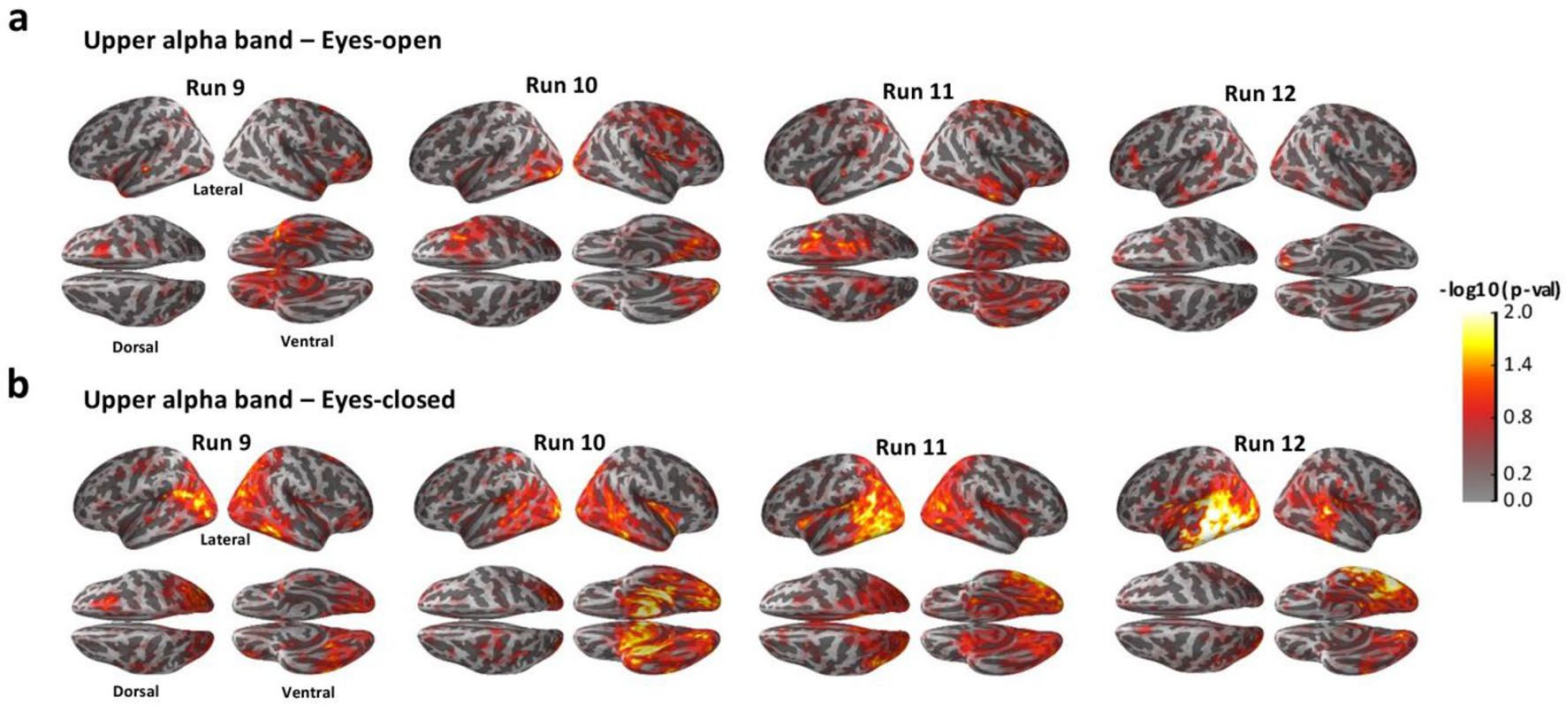
*p* value maps of one-way ANOVA on MEG baseline-corrected upper alpha band power spectral density of RF-EMF post-exposure sessions (real vs. sham) at source level analysis during eyes-open recordings (**a**) and eyes-closed recordings (**b**). *p* values were computed for each MEG source location (4098 current dipoles per hemisphere) for 29 subjects (*p* < 0.05). The cortex is shown inflated with gyri darker than sulci, with lateral (left and right), dorsal and ventral visualizations.

After MEG sensor space analysis, we focused on lower and upper alpha sub-bands, in the range of 8–10 Hz and 10–12 Hz, respectively, and we assessed their power spectral densities in source space. As for the sensor space data analysis, source space data were baseline-corrected and the two RF-EMF exposure conditions for lower and upper alpha band with EO and EC were compared (one-way ANOVA). Results are shown in Figs. 3 and 4 and resumed in Table 1 of Supplementary Information. Source space results were consistent with the results at sensor level.

Source space analysis revealed a statistically significant modulation in the real RF-EMF post-exposure relative to the sham in the power spectral density of lower alpha band with EO at temporal-parietal source locations from Run 10 to Run 12. The main effect was seen in the right frontal-parietal cortex during Run 11 (Fig. 3a). Lower alpha band in the EC condition was shown to be significantly modified in the real post-exposure relative to the sham at temporal-occipital cortical regions from Run 10 to Run 12 (especially during Run 12) (Fig. 3b). In particular, the left temporal lobe showed a greater number of source locations showing statistically significant power modulation than the right one, especially during the last recording run.

Upper alpha band spectral power with the EO condition was significantly modified after the real RF-EMF exposure at few source locations during the whole post-exposure recording period (Fig. 4a). In particular, during Run11 we observed the greatest number of sources showing statistically significant power modulation localized on the right fronto-parietal cortex. In the EC condition, upper alpha band spectral power was significantly modified after the real RF-EMF exposure from Run 9 to Run 12 (especially during Run 11 and 12) and this modulation affected a larger number of sources extended on temporal-occipital regions compared to the lower alpha band analysis (Fig. 4b). During Runs 11 and 12 the left hemisphere seemed to have more source locations significantly affected compared to the right hemisphere.

### Other physiological parameters

**Figure 5:**
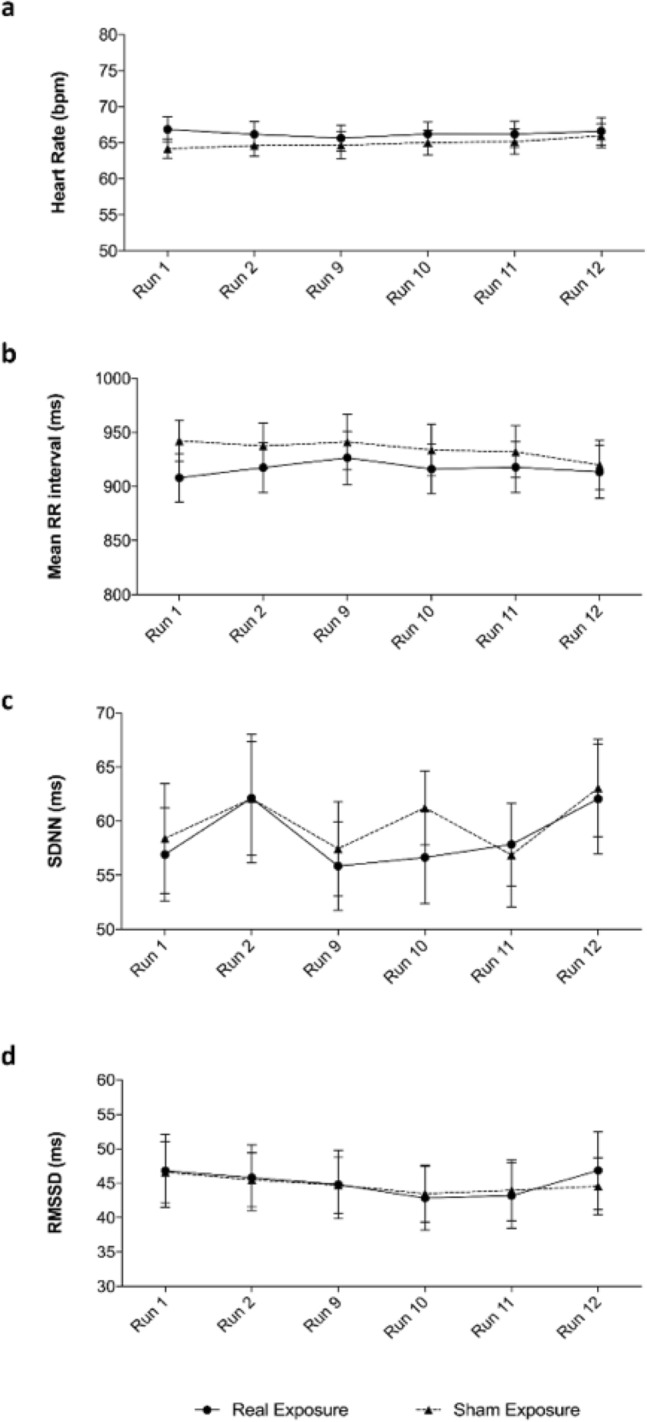
Results of ECG analysis. Heart rate data are show as means ± SEM (**a**). Heart rate variability data are show as means ± SEM and were estimated in time-domain: mean RR interval (**b**), standard deviation of the RR intervals, SDNN (**c**), square root of the mean squared differences between successive RR intervals, RMSSD (**d**).

Results of ECG analysis showed no significant difference in heart rate when comparing real to sham RF-EMF exposure sessions, no significant difference between runs and no significant interaction between factors (two-way ANOVA: F_1,36_ = 0.33, *p* = 0.56; F_2.42,87.27_ = 1.08, *p* = 0.35; interaction: F_5,180_ = 0.98, *p* = 0.43) (Fig. 5a). Results of the mean RR intervals showed no significant difference between real and sham RF-EMF exposure sessions, no significant difference between runs and no significant interaction between factors (two-way ANOVA: F_1,36_ = 0.32, *p* = 0.57; F_2.65,95.32_ = 1.25, *p* = 0.29; interaction: F_5,180_ = 0.87, *p* = 0.50) (Fig. 5b).

Standard deviation of the RR intervals (SDNN) analysis showed no significant difference between real and sham RF-EMF exposure session, no significant difference between runs and no significant interaction between factors (two-way ANOVA: F_1,36_ = 0.05, *p* = 0.82; F_3.39,121.9_ = 1.98, *p* = 0.11; interaction: F_5,180_ = 0.28, *p* = 0.92) (Fig. 5c). Square root of the mean squared differences between successive RR intervals (RMSSD) analysis showed no significant difference between real and sham RF-EMF exposure sessions, no significant difference between runs and no significant interaction between factors (two-way ANOVA: F_1,36_ = 0.00, *p* = 0.97; F_3.12,111.9_ = 1.92, *p* = 0.12; interaction: F_5,180_ = 0.32, *p* = 0.90) (Fig. 5d).

**Figure 6:**
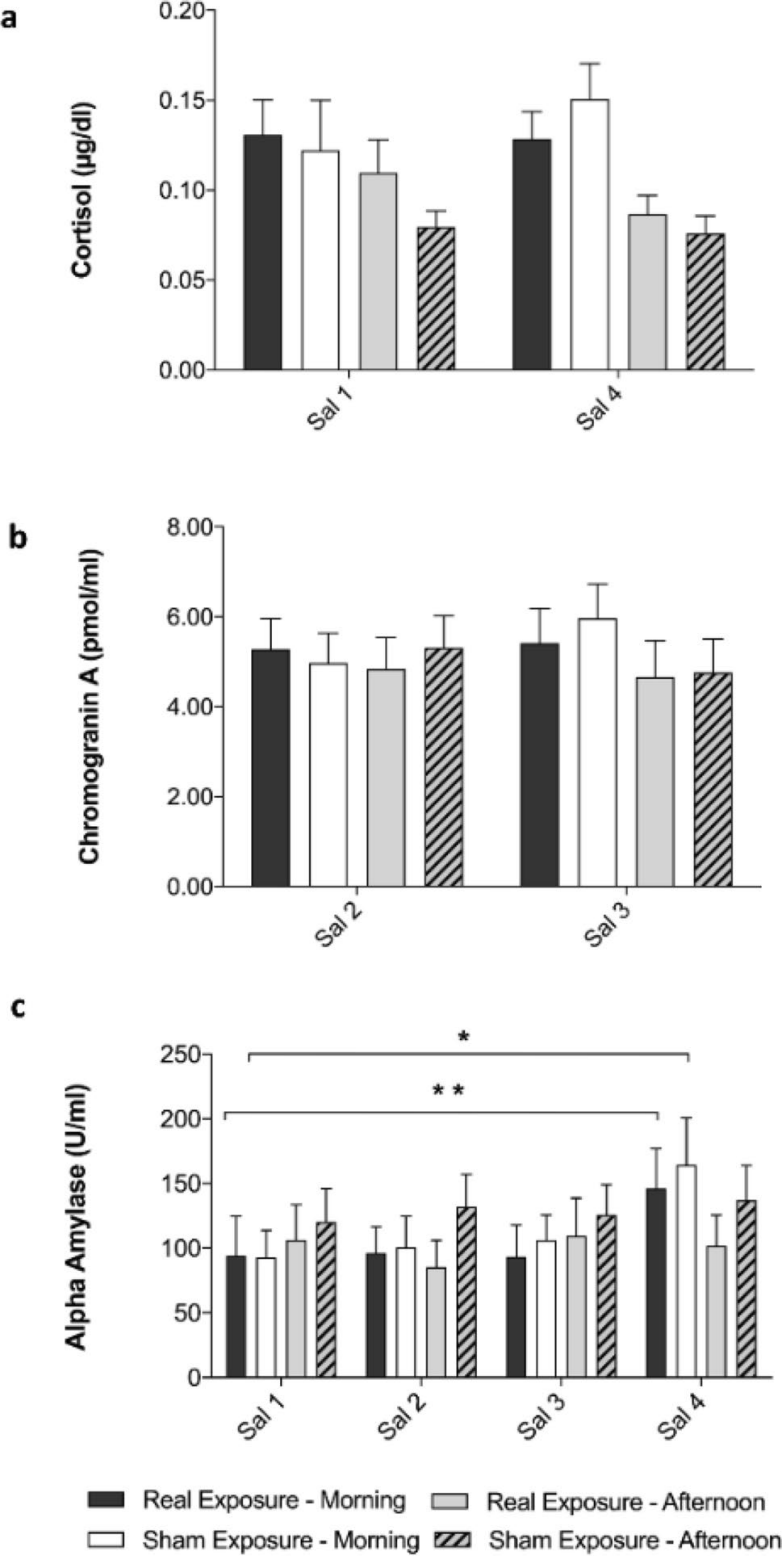
Results of biochemistry assays for salivary biomarkers of stress: cortisol (**a**), chromogranin A (**b**) and alpha amylase (**c**). Saliva samples (Sal) were analyzed considering samples collected during morning and afternoon experimental sessions, separately (13 and 16 subjects, respectively). Data are shown as means ± SEM. Statistical significance was set for *p* < 0.05 (**p < 0.01; *p < 0.05, one-way ANOVA followed by Bonferroni Multiple Comparison Test).

Results of biochemistry assays for salivary cortisol and chromogranin A collected in saliva samples in the morning and in the afternoon showed no significant differences in biomarker concentration (Fig. 6a,b).

When comparing real to sham RF-EMF exposure sessions, there were no significant differences between sessions and runs and no significant interaction between factors, as shown in Table 1.

**Table 1:** Statistical analysis results (F-values and *p* values) of biochemistry assays for salivary biomarkers of stress.

	Morning			Afternoon		
	Exposure condition	Time	Interaction	Exposure condition	Time	Interaction
Cortisol	F_1,24_ = 0.08 *p* = 0.78	F_1,24_ = 0.60 *p* = 0.45	F_1,24_ = 0.82 *p* = 0.37	F_1,30_ = 1.88 *p* = 0.18	F_1,30_ = 1.90 *p* = 0.18	F_1,30_ = 1.00 *p* = 0.32
Chromogranin A	F_1,24_ = 0.09 *p* = 0.77	F_1,24_ = 0.37 *p* = 0.55	F_1,24_ = 1.08 *p* = 0.31	F_1,30_ = 0.08 *p* = 0.78	F_1,30_ = 1.91 *p* = 0.18	F_1,30_ = 0.48 *p* = 0.50
Alpha Amylase	F_1,24_ = 0.01 *p* = 0.92	F_2.43,58.31_ = 6.94 ***p < 0.001	F_3,72_ = 0.23 *p* = 0.88	F_1,30_ = 0.71 *p* = 0.41	F_2.72,81.58_ = 0.40 *p* = 0.73	F_3,90_ = 1.07 *p* = 0.36

Saliva concentration of cortisol, chromogranin A and alpha amylase were analyzed by two-way repeated-measure ANOVA, considering as factors the exposure condition and the collection time in the morning or in the afternoon. For cortisol we considered time factor (two levels: Sal 1, Sal 4) and exposure condition factor (two levels: real exposure, sham exposure); for chromogranin A: time factor (two levels: Sal 2, Sal 3) and exposure condition factor (two levels: real exposure, sham exposure); for alpha amylase: time factor (four levels: Sal 1, Sal 2, Sal 3, Sal 4) and exposure condition factor (two levels: real exposure, sham exposure). Only alpha amylase collected in the morning showed a statistically significant variation, due to time factor (***p < 0.001).

Alpha amylase salivary concentration showed a statistically significant increase related to time factor, only during the morning experimental sessions (Table 1). A further one-way ANOVA analysis was run followed by Bonferroni Multiple Comparison Test to further detect differences between alpha amylase saliva samples Sal 1, Sal 2, Sal 3 and Sal 4 collected in the morning. The Bonferroni Multiple Comparison Test revealed a statistically significant increase in morning alpha amylase concentration between Sal 1 and Sal 4 for both real (*p* < 0.01) and sham (*p* < 0.05) RF-EMF exposure sessions (Fig. 6c).

Results of HPLC to determine caffeine levels in saliva showed only five subjects with caffeine quantification greater or equal to the limit of quantification set at 0.5 µg/ml. For one subject caffeine concentration was measured at 0.7 µg/ml at the real exposure session, for three subjects it was measured at 0.7 µg/ml, 1.1 µg/ml and 1.8 µg/ml at the sham exposure session and for another one it was measured at 0.5 µg/ml and 0.7 µg/ml at real and sham exposure sessions, respectively.

## Discussion

To our knowledge, this study is the first to use sensor and source space of MEG to analyze the effect of 900 MHz RF-EMF exposure on the spontaneous alpha band activity of healthy volunteers in a crossover, randomized, double-blind and counterbalanced experimental design. Alpha band power spectral densities were calculated at each sensor and source location.

Observed results indicated a significant effect of 900 MHz RF-EMF exposure on the alpha band MEG activity detected in sensor and source space, mainly represented as a decrease of spectral power amplitudes. In particular, EO resting-state sensor analysis revealed a modification of the entire alpha band and both frequency sub-bands after RF-EMF exposure, especially at the parietal cortex. These results were confirmed by analysis at source level, showing an alpha band modulation widespread at the fronto-parietal region. Otherwise, with the sensor space analysis of MEG recordings in EC condition, results showed a decrease of the entire alpha band power and the upper alpha power after RF-EMF exposure, especially at the temporo-occipital region. The results were supported by source space analysis, which also highlighted the power modulation of the lower alpha band.

As widely reported in the literature, alpha band spectral power at rest is topographically characterized by greatest amplitude at posterior temporal-occipital and parietal regions during resting-state, especially when eyes are closed^54^. Indeed, alpha power desynchronizes as a consequence of eyes opening^54^. For this reason, we can assume that we found a greater RF-EMF effect on alpha MEG spontaneous activity at posterior regions when participants closed their eyes, rather than with EO. Furthermore, during the EC condition the power modulations was mainly localized posteriorly and on the left temporal lobe, as confirmed by source space analysis. Since the MP was placed against the left ear during the exposure phase for about 26 min, we could assume a lateralization of the RF-EMF effect related to the exposed head side (i.e. ipsilateral effect). Similarly, EEG alpha band power has been shown to decrease in healthy volunteers during 20 min of extremely low frequency exposure, with an effect detected only on the exposed head side^55^. Other ipsilateral RF-EMF effects have been seen on the regional cerebral blood flow, which was found to be increased at dorsolateral prefrontal ipsilateral cortex in positron emission tomography scans after 900 MHz GSM signal exposure^56^, and on cortical excitability of focal epileptic patients, which showed cortical excitability increasing on the ipsilateral hemisphere using transcranial magnetic stimulation after GSM MP exposure^57^. Moreover, GSM MP exposures have been shown to modulate the inter-hemispheric coherence of temporal and frontal resting EEG alpha rhythm in both young and elderly populations^26,58,59^. It is noteworthy that previous dosimetry studies showed that the temporal lobe presented the highest average relative SAR with RF-EMF frequencies between 900 MHz and 1800 MHz with MP placed in ipsilateral phantom cheek position^48^. Furthermore, the average relative SAR has been showed to decrease with increasing depth in brain anatomical layers, particularly at higher frequencies^48^.

On the other hand, our results showed that when participants were recorded with their eyes open staring a fixation point localized 290 cm from the gaze, alpha band spectral power was mainly affected by RF-EMF exposure at the fronto-parietal region, with a greater number of sources showing a significant modulation located on the contralateral right hemisphere. It is noteworthy that previous functional MRI studies showed that the ocular motor system and the attentional system were more activated with eyes-open condition^60^. In particular, several cortical areas of the fronto-parietal lobes were involved with the ocular motor system activity in gaze control (i.e., supplementary eye field, frontal eye field, parietal eye field)^61^, and sustained attention seemed to be predominately related to the fronto-parietal area of the right hemisphere^60^. Furthermore, different patterns of desynchronization can be observed considering from the entire alpha band both the lower and the upper alpha sub-bands, separately^62^. For this reason, we deemed necessary to analyze the MEG alpha band activity splitting the entire 8–12 Hz band into 8–10 Hz and 10–12 Hz sub-bands. Indeed, previous studies showed that in the EO condition the upper alpha band in the range of 10–14 Hz could synchronize in cortical networks at frontal, parietal and visual regions as a consequence of visuospatial attention^63^. Otherwise, during the visuospatial attention task the spectral power of the lower alpha band in the range of 6–9 Hz resulted in suppression in the posterior visual cortex^63^, while general task demands and attentional processes determine lower alpha desynchronization between 6 and 10 Hz widespread over the scalp^62^. Taken together, our results suggested that it was easier to appreciate the RF-EMF effect on alpha MEG activity at cortical regions where alpha oscillations (both lower and upper) were dominant when subjects closed or opened their eyes.

Since the MEG system is sensitive to any kind of electromagnetic source, we could record the MEG cerebral activity only after the MP exposure period. Anyway, thanks to different recording blocks (i.e. Run 9, Run 10, Run 11 and Run 12) during the post-exposure phase of the experimental protocol, we could follow the time course of radiofrequency effects across runs after the exposure. So, with the aim to better appreciate any modulation of the MEG power after the RF-EMF exposure, we did not average the MEG power values across the post-exposure runs before our statistical analysis. MEG post-exposure phase recordings started about 4 min after the MP removing from the left side of the head, which followed the transition of the subject from the EEG shielded room to the MEG one with a wheelchair. Both the real and the sham exposure sessions were characterized by the same experimental protocol, and we could therefore exclude possible differences in MEG activity between sessions related to the passage between two rooms. It is noteworthy that during Run 11 and Run 12 we observed a greater number of sources showing power modulation for both lower and upper alpha bands related to the real exposure compared to other runs. We could argue that a delay period was necessary to restore resting-state and alpha oscillations synchronization after the installation in the MEG shielded room and post-exposure phase starting. Furthermore, a delay of 15 min in the onset of EEG activity modulation after exposure has been suggested^23^. All together these findings on MEG spontaneous activity not only seemed to confirm our previous study^17^, which showed a widespread decrease of the entire alpha band (8–12 Hz), lower and upper alpha sub-bands spectral power of resting spontaneous EEG with EC condition during and after 900 MHz GSM RF-EMF exposure, but also found slight effects of RF-EMF exposure on alpha frequency in the EO condition. These results were also in line with other studies showing a decrease of alpha band spectral power during GSM signal exposure, with an effect localized on the whole scalp^16^ or mainly at the occipital region^24,25^. Moreover, similar results were found during 3G and 4G RF-EMF signals exposure with a global alpha band modulation (i.e. power decrease) over the scalp^27^ or mainly localized at the fronto-temporal contralateral region^26^. These differences in the alpha band topographical modulation at rest are probably due to differences in the position of the exposure system and the number and distribution of EEG electrodes considered for statistical analysis^7^. On the other hand, other studies revealed an increase of the alpha band power during RF-EMF exposure^14,15,18–22^. Globally, we suggest that these discrepancies in results could be related to different experimental protocols^6,7^ and also to individual variability^21^.

The measurements of heart rate, HRV, biomarkers of stress and caffeine analysis mean that we could exclude the possibility that our results were biased by confounding factors, such as stress behavior or caffeine consumption before the experiment. Indeed, none of these parameters showed a significant difference between experimental sessions and runs. In particular, ECG data analysis results were in line with reference ranges for short-term measures of HRV in healthy adult populations^50^. The salivary free cortisol concentration (a reliable marker of HPA system activity that correlates with serum adrenocorticotropin with a 15-min delay^51^), was analyzed at the beginning and at the end of each experimental session. We further monitored any stress-related changes in the autonomic nervous system via the sympatho-adreno-medullary system. To this aim, the level of salivary chromogranin A (secretion of which correlates with sympathoadrenal release of catecholamines^64^) was measured before exposure phase and post-exposure phase beginnings. Finally, since alpha amylase is directly secreted by salivary glands under the sympathetic regulation^53^, we widely assessed its concentration during the whole duration of each experimental session. Only the alpha amylase from saliva samples collected during real and sham morning sessions, indicated a significant increase related to the time factor. We assume that this variation was caused by its physiological circadian rhythm variation^53^. Moreover, results of HPLC analysis revealed a negligible caffeine concentration in saliva samples of all participants at the beginning of the experimental sessions, with the maximum concentration assessed at 1.8 µg/ml.

Even though consistent evidence suggests that RF-EMF can affect spontaneous alpha activity^6^, the reason why the alpha oscillation reacts differently from other frequency bands is still unclear^27^. We can assume that the majority of studies carried out in this field found a modulation of the alpha band because of its great reliability. In fact, EEG test–retest studies showed that alpha and beta bands were the most reliable frequency bands^65,66^. In addition, power in the EC EEG resting-state condition was shown to be more reliable than the EO condition^67,68^. Alpha band also confirms its great power reliability in a MEG study at rest in sensor and source space and, interestingly, sensor space power at the posterior region presents higher reliability in the EC condition than the EO condition^69^. This evidence could further explain the main modulation of lower and upper alpha bands that we found after RF-EMF exposure at the temporal-occipital region whilst subjects closed the eyes. Since alpha band showed great reliability in common functional connectivity estimation with MEG^70^, future studies on resting MEG functional connectivity could help to understand the effect of RF-EMF exposure on the neuronal cortical network defining the correlation between source locations, especially with regard to the side of the exposure.

In conclusion, using a MEG sensor and source space analysis our study, to the best of our knowledge, confirmed for the first time that about 26 min of 900 MHz exposure can modulate both lower and upper alpha bands at rest with an effect detectable during the post-exposure period. This modulation in sensor and source space was mainly represented by a decrease in both frequency alpha sub-band power spectral densities at parietal and temporal-occipital regions and depended on whether eyes were open or closed. We may suppose that these changes in MEG cortical activity may reflect the neurophysiological modifications (i.e. altered membrane potential and neuronal oscillations) induced by non-thermal exposure to RF-EMF as consequence of different water polarization and diffusion processes^35,36^. Finally, future investigations on MEG functional connectivity could be helpful to further understand the electrophysiological dynamics which determine the RF-EMF modulation of the alpha band.

## Subjects and methods

### Participants

Thirty-two right-handed healthy volunteers with normal or corrected to normal vision were recruited (15 males and 17 females, mean ± SD age: 24.8 ± 3.5 years, mean ± SD body-mass index: 21.61 ± 1.87). They were selected according to strict inclusion criteria. None of the participants had any previous history of head injury, neurological or psychiatric disease or any chronic disease, disability or recent acute illness during last month. Pregnant women were not included in the study (confirmed by urine pregnancy test, NADAL hCG Pregnancy test 10 mIU/ml, nal von minden GmbH, Germany). Inclusion criteria also included regular sleep habits with a regular wake time (8 a.m.–11 p.m. ± 1 h), no medication, non-smokers and no-drug use. No-drug use was confirmed by a urine multi-drugs test (NarcoCheck Evolutive, France), in order to detect tetrahydrocannabinol, cocaine, crack cocaine, opiates and opioids (morphine, heroin, fentanyl, methadone, buprenorphine), ketamine, lysergic acid diethylamide, alpha-methylphenethylamine, 3,4-methylenedioxymethamphetamine, benzodiazepines, barbiturate and tricyclic antidepressants. Participants were asked to abstain from consuming alcohol and caffeine for 24 h before each session and to fast for at least two and a half hours before the beginning of the recording session. They were asked to maintain a regular wake time (8 a.m.–11 p.m. ± 1 h) at least one week before the experiment and not to use their personal MP on the day of the experiment. Only women with regular menstrual cycles of 26–32 days for the preceding 6 months were included. They participated only during the follicular phase of their ovarian-menstrual cycle five days after the first day of the menses phase. All participants provided informed written consent and were compensated for their participation. Informed consent was obtained to publish images in an online open access publication. All procedures were approved by the French Ethics Committee CPP Ouest VI (ID number: RCB 2017-A01702-51) and were in accordance with the Declaration of Helsinki.

### Exposure system

**Figure 7:**
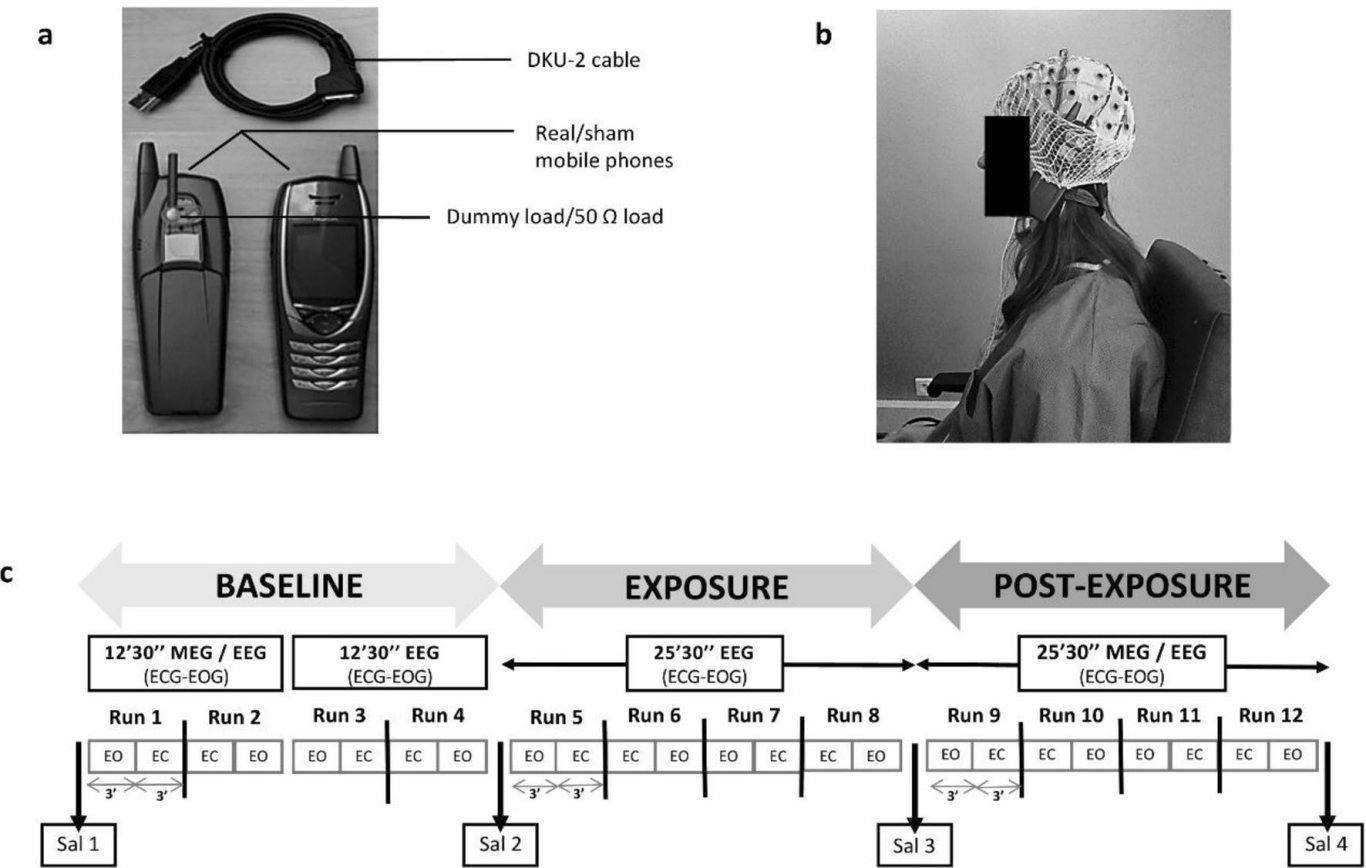
Exposure system (**a**), exposure setup (**b**) and experimental protocol (**c**). Both mobile phones looked the same, but their electromagnetic radiation field was different. One of them was placed against the left ear with a tubular tissue bandage during the exposure. Each recording session included three phases: the baseline phase, with 12 min and 30 s of magnetoencephalography (MEG) and electroencephalography (EEG) simultaneously recorded and 12 min and 30 s of EEG recording; the exposure phase with 25 min and 30 s of EEG recording; the post-exposure phase with 25 min and 30 s of MEG and EEG combined recordings. Recording runs were performed with 3 min of eyes-open (EO) and eyes-closed (EC) sequences in alternation. After each run there was 30 s pause. Electrocardiogram (ECG) and electro-oculogram (EOG) were simultaneously recorded. Four saliva samples were collected (Sal 1, Sal 2, Sal 3, Sal 4).

In order to ensure a double-blind design, the exposure systems were two commercial dual-bands GSM MPs (Nokia 6650) with exactly the same shape and structure, which differed only in the RF-EMF emission, as previously reported^3,17^. Briefly a 50 Ω resistive load and an open-circuit dummy load were connected to the external antenna connector of the sham and the real MP, respectively (Fig. 7a). Before starting the exposure phase, the MPs were connected to a personal computer with a DKU-2 cable by an independent researcher in order to set the frequency of interest and RF-EMF power using service software (Phoenix, Nokia, Finland). The emitted signal was a GSM signal working at a frequency of 900 MHz, modulated at 217 Hz with a maximum power of 2 W, equivalent to an average power of 250 mW with 1/8 duty cycle. The maximum SAR averaged on 10 g tissue, 1 g tissue and the peak value were measured at 0.49 W/kg, 0.70 W/kg and 0.93 W/kg, respectively. The SAR of the sham phone was below the detection level of the system (0.001 W/kg) at any position of the phantom and no electric field was detected on the surface of the sham phone, as previously reported^3^. To start the exposure, the real or the sham MP was disconnected from the personal computer and placed against the left ear with a tubular tissue bandage during the whole time of the exposure phase (Fig. 7b).

### Experimental protocol

Participants attended two recording sessions one week apart at the same time of the day, to avoid possible interference with circadian rhythm. We used a crossover, randomized, double-blind and counterbalanced experimental design. Each session was characterized by three recording phases: the baseline with no RF-EMF exposure, the exposure period with real or sham RF-EMF exposure and the post-exposure period with no RF-EMF exposure. Because of the interference of the RF-EMF on MEG sensors, the MEG was recorded only during baseline and post-exposure phases in a specific soundproofed shielded room (MaxShield System, Elekta Neuromag, Helsinki, Finland). Conversely, the EEG was recorded throughout the duration of the experimental session in another soundproofed electrically shielded room. EEG data will not be reported in this paper.

Recording phases started at about 11.30 a.m. or 3.30 p.m. for each experimental session scheduled with a counterbalanced design in the morning or in the afternoon, respectively (finally thirteen volunteers took part to morning sessions). At the beginning of the recording block, subjects were instructed to stay still, to stare a fixation point (a black cross placed 290 cm from the subject in the MEG room) and to stay relaxed and awake. They were monitored in real-time via a camera in order to verify their vigilance and adherence to instructions during recording runs. All participants respected the instructions during both experimental sessions. We did not remark any decrease of vigilance, excepting for two subjects who showed sleep onset especially during recording blocks with eyes open. They interrupted their participation after the first experimental session.

The baseline included 12 min and 30 s of MEG and EEG simultaneously recorded (Run 1 and Run 2) followed by 12 min and 30 s of EEG (Run 3 and Run 4). The exposure phase lasted 25 min and 30 s with only EEG recordings (from Run 5 to Run 8). The post-exposure phase lasted 25 min and 30 s with MEG and EEG simultaneously collected (from Run 9 to Run 12) (Fig. 7c). During the baseline phase, we applied just two runs per technical approach, in order to limit the total duration time of the protocol, as it could become too wearisome to bear for participants. On the other hand, we extended the exposure and post-exposure phases in order to better follow the time course of potential effects after MP exposure. The passage from one shielded room to another was done with a wheelchair to limit motor activity. The MP was removed just before the entrance to the MEG shielded room and the post-exposure recording started after 3 min and 54 s ± 61 s from the MP removal. Each run was characterized by the alternation of 3 min EO and 3 min EC sequences. After each 6 min there was a pause of 30 s. At the beginning of each phase and at the end of the session, a salivary sample was collected with a Salivette device (Sarstedt, Germany) making a total of four saliva samples (Sal 1, Sal 2, Sal 3 and Sal 4) (Fig. 7c).

A final visit was scheduled for each participant after the recording sessions in order to provide an anatomical cerebral MRI, acquired with T1-weighted MPRAGE sequence (Siemens, Magnetom, Verio 3 T).

### Data acquisition

Continuous MEG cortical signals were collected using a whole-head MEG system with 102 magnetometers and 204 planar gradiometers (Elekta Neuromag TRIUX MEG system, Helsinki, Finland) at a sampling rate of 1000 Hz with DC recordings and on-line lowpass filtered at 330 Hz.

To localize the head before each acquisition, four Head Position Indicator (HPI) coils were attached onto the EEG cap. The EEG cap was compatible with MEG system, thanks to 74 electrodes which were MEG-suited with sintered Ag/AgCl sensors (Brain Products GmbH, Herrsching, Germany). The 3D locations of HPI coils, three cardinal landmarks (nasion, left and right periauricular), EEG electrodes and additional points on the scalp were digitized using a Fastrak system (Polhemus, Colchester, VT, USA). Horizontal electro-oculogram (EOG) was acquired by placing one electrode at each canthus of the eyes, while vertical EOG was acquired by placing one electrode below and another above the right eye. Electro-cardiogram (ECG) was recorded with an electrode on the right clavicle and one on the left lower hip region. EOG and ECG were recorded during experimental sessions with a bandwidth of 0.1–330 Hz.

### MEG data pre-processing

Firstly, head positions during MEG recordings were averaged between runs and sessions for each participant. Temporal Signal Space Separation (tSSS) with averaged head position was performed using MaxFilter (Elekta Neuromag) to remove external noises and to get a homogeneous MEG sensor space signal across the runs. A visual inspection was made to remove data epochs contaminated by MEG sensors jumps. If jumps occurred frequently along the signal, the corresponding sensors were removed and interpolated with the neighboring ones. Further data analysis was carried out using the MNE-Python software package^71^, version 0.16. MEG data were band-pass filtered with Finite Impulse Response (FIR) filter design using the Hamming window method (Hamming window with 0.0194 passband ripple and 53 dB stopband attenuation). The lower passband edge was set at 1 Hz with 1 Hz transition bandwidth (− 6 dB cut-off at 0.50 Hz) and the upper passband edge was at 30 Hz with 7.50 Hz upper transition bandwidth (− 6 dB cut-off at 33.75 Hz). Ocular and cardiac artefacts were corrected on MEG data (both gradiometers and magnetometers) using the Independent Component Analysis (ICA) with FastICA algorithm. We decimated the data by a factor of 3 and excluded time segments exceeding amplitude ranges of 4000 × 10^−13^ T cm^−1^ and 5 × 10^−12^ T on gradiometers and magnetometers, respectively. Dedicated EOG and ECG sensors were used as a pattern to check the independent components and to automatically select and reject the corresponding artifact components from the decomposition. Pearson correlations were used to find EOG related components while independent components corresponding to ECG activity were identified using cross-trial phase statistics applying a threshold of 0.25 on the Kuiper statistic components. Bad epochs were created 500 ms before and after each blink and heartbeat event, with band pass filtering between 1 Hz and 10 Hz and 8 Hz and 16 Hz, respectively.

### MEG time frequency analysis

Fast Fourier Transformation (Welch technique, 512 points per window, Hamming window with no overlap) was applied on ICA-corrected magnetometer and gradiometer data, considering each baseline and post-exposure run during EO and EC periods separately. Power spectral density was computed for the entire alpha band in the range of 8–12 Hz and for both lower and upper alpha sub-bands in the range of 8–10 Hz and 10–12 Hz, respectively. This approach allowed us to observe any distinct pattern of the modulation of the extended alpha band, according to Klimesch et al.^62^. Since individual alpha frequency peak lies in the range of about 9.5–11.5 Hz for young healthy adults^62^, and in order to limit inclusion of other frequency bands in the power spectral density analysis, we restrain the frequency interval to 8–12 Hz for all subjects. The log-transformation of the data was used to approach a normal distribution. For the purpose to combine together the orthogonal planar gradiometers, their logarithmic power values were averaged. Finally, logarithmic power values of baseline recordings Run 1 and Run 2 were averaged together, considering EO and EC conditions separately.

### MEG source space analysis

Anatomical MRI images of each subject were automatically segmented using the FreeSurfer software package^72^, version 6.0.0. A co-registration step allowed positioning of the MRI images and the sensors in a common coordinate system, using HPI and cardinal landmarks. The source model was built with 4,098 current dipoles per hemisphere distributed on the surface of the white matter and normally oriented to the local cortical surface, yielding a distance of 4.9 mm between neighboring points. The forward model was estimated using the boundary element model (BEM) computed on the inner skull with 0.3 S/m of conductivity. Whitening data with noise covariance estimation enabled us to scale the magnetometers and gradiometers at the same unit. Sources were estimated with the Minimum Norm Estimate (MNE) inverse operator. Finally, the power spectrum density was computed (512 points per window, in dB) across all subjects, in each session, run and EO and EC condition. According to our previous MEG sensor space analysis, lower and upper alpha sub-bands at 8–10 Hz and 10–12 Hz, respectively, were considered. To allow statistical comparison between subjects, current estimates from all participants were morphed to a common template brain.

### Heart rate and heart rate variability

In order to analyze the heart rate and HRV, the RR normalized intervals were acquired from 5 min of ECG signal recorded during each run of the baseline and the post-exposure phases. Heart rate was measured detecting heartbeat peaks from dedicated ECG channels for each run (MNE-Python software package, version 0.16). With the purpose of realizing short term HRV analysis^50^, ten subjects were excluded from further ECG analysis because the algorithm did not locate QRS component in ECG segments shorter than 1.2 s^73^ for a total time greater or equal to 30 s in one or more runs of experimental session. The heart rate was calculated according to the formula: 60/RR intervals. HRV was estimated considering the following time domain parameters: mean RR intervals, SDNN and RMSSD.

### Biochemistry assays for biomarkers of stress

Saliva samples collected with Salivette devices were immediately stored at 4 °C. Within 24 h, the samples were centrifuged (1000 g/20 min/4 °C) and the supernatant was collected and immediately frozen at − 80 °C with aliquots. Samples were stored without preservatives until analysis. Cortisol was quantified in the samples Sal 1 and Sal 4 using commercialized competitive enzyme immunoassay kits for human salivary cortisol, according to the manufacturer’s instructions (Salivary Cortisol EIA kit, Salimetrics, LLC). Chromogranin A was quantified in samples Sal 2 and Sal 3 using commercialized competitive enzyme immunoassay kits for human salivary Chromogranin A, according to the manufacturer’s instructions (Chromogranin A Human EIA kit, Yanaihara Institute Inc., Japan). Alpha amylase was quantified in samples Sal 1, Sal 2, Sal 3 and Sal 4 using commercialized liquid phase enzymatic assay for human salivary alpha amylase, according to the manufacturer’s instructions (Alpha Amylase Saliva Assay, IBL International, Hamburg, Germany). All samples for all parameters were tested in duplicate and the average of duplicates was used as the final value.

### High-performance liquid chromatography for caffeine concentration

High-performance liquid chromatography (HPLC) method was used to quantify caffeine concentration in saliva sample Sal 1 for each experimental session for each subject. Chromatography was performed on Agilent 1200 Series Gradient HPLC System (Agilent Technologies, Inc., CA) consisting of a quaternary gradient pump, standard autosampler, column thermostat and variable wavelength detector. An Envirodur C18 column (250 × 4.6 mm, 3 µm; Macherey Nagel) was used for the separation. The mobile phase was made of 85% of a 0.012 M KH_2_PO_4_ and 15% acetonitrile. The flow rate was set at 1.0 ml/min and the injection volume was set at 20 µl. The detection wavelength was set at 280 nm. The limit of quantification was set at 0.5 µg/ml. The caffeine solution concentrations used for the standard curves were 0.1, 1.0, 5.0 and 10 µg/ml. Standard curves were constructed by plotting concentration vs. area under the curve. Caffeine retention time was 3.4 min.

### Statistical analysis

Since two volunteers participated only in the first recording session and one participant showed large artefacts on MEG after tSSS, a total of 29 subjects were considered for statistical analysis. The exclusion of three volunteers affected just partially the counterbalanced design, which involved two possible exposure sequences (fourteen volunteers had a real exposure at their first session; thirteen volunteers participated at experimental sessions in the morning).

Statistical analysis was performed using Matlab-based in-house toolbox for statistical analysis of MEG-EEG data^74^. Log-transformed power values were used. Firstly, a 2 × 2 × 2 repeated-measures ANOVA with time (two levels: baseline, post-exposure), eyes condition (two levels: EO, EC) and exposure (two levels: real, sham) as factors was applied to power spectral densities computed on sensor space data for the entire alpha band and for both lower and upper alpha sub-band across magnetometers and gradiometers. Baseline Run 1 and Run 2 were previously averaged together, considering EO and EC conditions separately.

Secondly, and in order to characterize the exposure effect on alpha frequencies, the averaged baseline power was subtracted from the power of each post-exposure run. One-way ANOVA on baseline-corrected data was applied to determine the effect of RF-EMF exposure on alpha band and lower and upper alpha sub-bands during EO and EC sequences. The real post-exposure power values computed at each MEG sensor were compared with the sham post-exposure ones to compute F and *p* values for each sensor element.

Finally, as with the MEG sensor data, power spectral densities computed in source space for baseline data of Run 1 and Run 2 were averaged together. Source power of lower and upper alpha frequencies of post-exposure runs with EO and EC were baseline-corrected and analyzed with one-way ANOVA, comparing the two exposure conditions and computing F and *p* values for each source location.

To control for multiple comparisons (multiple sensors), the conducted parametric hypothesis tests were followed by cluster based permutation. This method was proposed by Maris et al.^75^ and implemented in Fieldtrip for t-test. We extended it to repeated measures ANOVA^74^. For each main effect or interaction, samples whose F-value exceeded a threshold (here, F-values corresponding to a one-tail *p* value of 0.05) were clustered based on space adjacency given by a sensor neighboring matrix. Each space-defined cluster is assigned a cluster-based statistic equal to the sum of the F-values of all the samples belonging to the cluster. Then, the condition labels of the individual data are randomly shuffled. The clustering procedure is applied on those randomized data, and the maximal cluster F-value is calculated for each factor and interaction. The distribution of the maximum cluster level F-statistics under the null hypothesis is estimated through 1,000 randomizations for each main effect and each possible interaction. The null hypothesis is rejected with a Monte-Carlo *p* < 0.05 if the observed statistic is greater than 95% of the statistic values obtained from randomized data.

Heart rate and HRV data analyses were performed using a two-way repeated measure ANOVA, followed by Bonferroni adjusted post hoc analysis. The factors were: time factor (six levels: Run 1, Run 2, Run 9, Run 10, Run 11, Run 12) and exposure condition factor (two levels: real exposure, sham exposure). Non-sphericity was assessed with Geisser-Greenhouse correction. Results of biomarkers of stress quantification were analyzed using a two-way repeated measure ANOVA followed by Bonferroni adjusted post hoc analysis. Participants recorded in the morning and in the afternoon were considered separately. For cortisol there were two factors: time factor (two levels: Sal 1, Sal 4) and exposure condition factor (two levels: real exposure, sham exposure); for chromogranin A there were two factors: time factor (two levels: Sal 2, Sal 3) and exposure condition factor (two levels: real exposure, sham exposure); for alpha amylase there were two factors: time factor (four levels: Sal 1, Sal 2, Sal 3, Sal 4) and exposure condition factor (two levels: real exposure, sham exposure). Non-sphericity of alpha amylase data was assessed with Geisser-Greenhouse correction. All physiological parameters data were analyzed and plotted with GraphPad Prism, version 8. Statistical significance was set for *p* < 0.05.

## Supplementary Information

Supplementary Figure 1.

Supplementary Figure 2.

Supplementary Table 3.
