# Supplementary material for: Modulation of magnetoencephalography alpha band activity by radiofrequency electromagnetic field depicted in sensor and source space
Source: Sci Rep. 2021 Dec 3;11:23403. doi: 10.1038/s41598-021-02560-0 (PMC8642443; doi:10.1038/s41598-021-02560-0)
Supplement: Supplementary file 1 — Supplementary Figure 1. [file 41598_2021_2560_MOESM1_ESM.docx]

**
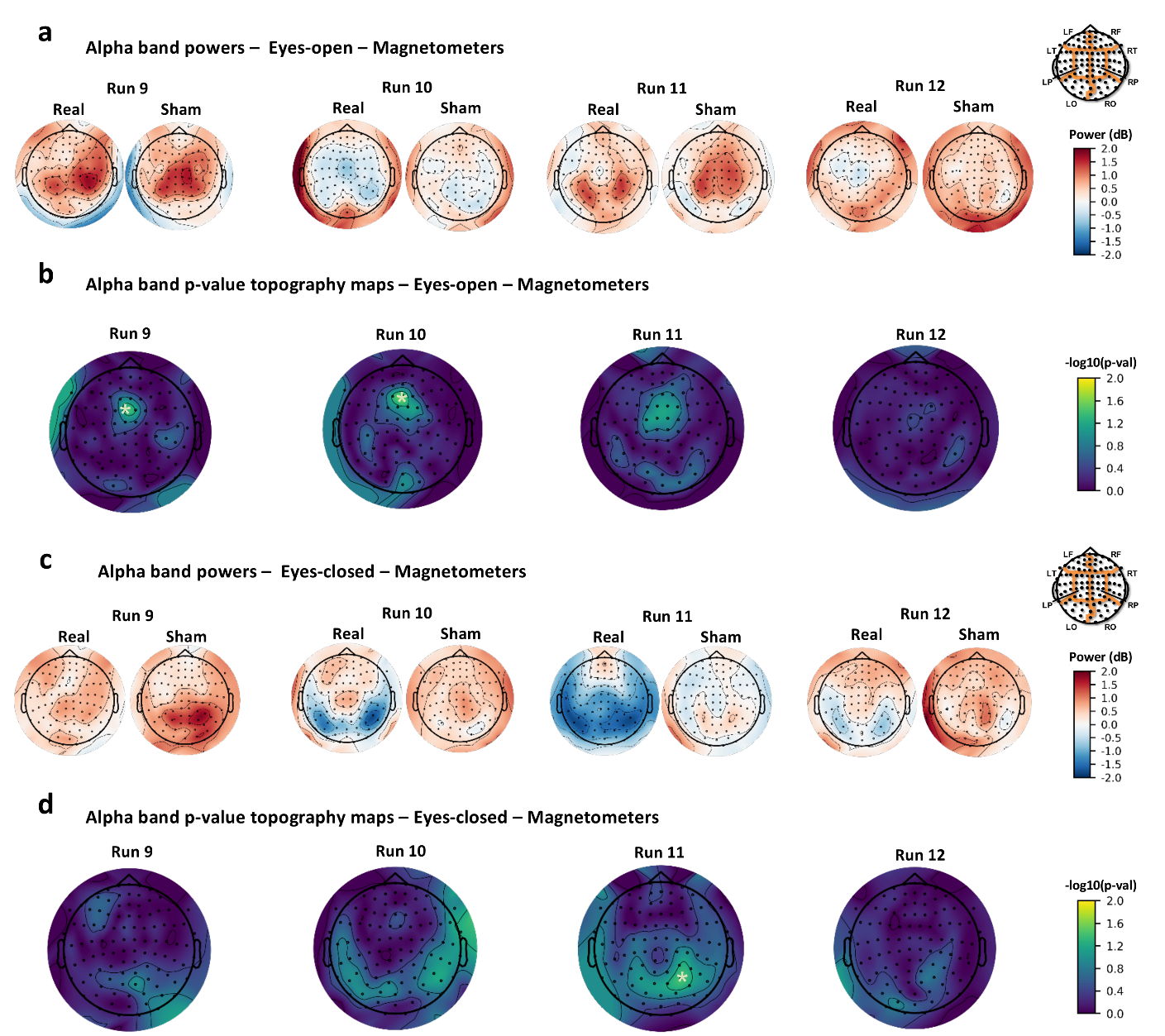
**

**Figure 1 supplementary information. MEG sensor space results for the entire alpha band (8–12 Hz) with magnetometers during eyes-open recordings (a, b) and eyes-closed recordings (c, d).** Power topography maps show baseline-corrected power values computed in sensor space for each sensor during runs of the real and the sham exposure session for 29 subjects and plotted according to the 102 MEG sensor triplets layout (LF, left frontal region; RF, right frontal region; LT, left temporal region; RT, right temporal region; LP, left parietal region; RP, right parietal region; LO, left occipital region; RO, right occipital region). P-value topography maps show the results of one-way ANOVA on MEG baseline-corrected entire alpha band power of RF-EMF post-exposure sessions (real vs. sham) at sensor level analysis. P-values were computed for each sensor for each run (*p<0.05).
