# Supplementary material for: Modulation of magnetoencephalography alpha band activity by radiofrequency electromagnetic field depicted in sensor and source space
Source: Sci Rep. 2021 Dec 3;11:23403. doi: 10.1038/s41598-021-02560-0 (PMC8642443; doi:10.1038/s41598-021-02560-0)
Supplement: Supplementary file 3 — Supplementary Table 3. [file 41598_2021_2560_MOESM3_ESM.docx]

**Table 1 supplementary information.** MEG sensor space results of one-way ANOVA on MEG baseline-corrected power (Real post-exposure phase vs. Sham post-exposure phase) computed for the entire alpha band (8–12 Hz) and lower (8–10 Hz) and upper (10–12 Hz) alpha sub-bands during eyes-open or eyes-closed recordings in sensor space and in source space.

|  | **Sensor space** | | | | | | | | **Source space** | | | |
| --- | --- | --- | --- | --- | --- | --- | --- | --- | --- | --- | --- | --- |
|  | **Magnetometers** | | | | **Gradiometers** | | | |  |  |  |  |
|  | ***Eyes-open*** | | ***Eyes-closed*** | | ***Eyes-open*** | | ***Eyes-closed*** | | ***Eyes-open*** | | ***Eyes-closed*** | |
|  | ***F-P*** | ***T-O*** | ***F-P*** | ***T-O*** | ***F-P*** | ***T-O*** | ***F-P*** | ***T-O*** | ***F-P*** | ***T-O*** | ***F-P*** | ***T-O*** |
| ***8–12 Hz*** | Power decreased during Run 9 and 10 | NS | NS | Power decreased during Run 11 | Power decreased during Run 11 | NS | NS | Power decreased during Run 11 and 12 | NR | NR | NR | NR |
| ***8–10 Hz*** | Power decreased during Run 10 and 11 | NS | NS | NS | Power decreased during Run 11 | NS | NS | NS | Power modulation during Run 10 and 11 | Power modulation during Run 10, 11 and 12 | NS | Power modulation during Run 10, 11 and 12 |
| ***10–12 Hz*** | Power decreased during Run 9 and 10 | Power increased during Run 10 | Power decreased during Run 11 | Power decreased during Run 10 and 11 | NS | NS | Power decreased during Run 9 and 11 | Power decreased during Run 10, 11 and 12 | Power modulation during Run 10 and 11 | Power modulation during Run 9 and 10 | NS | Power modulation during Run 9, 10, 11 and 12 |

F-P, fronto-parietal

T-O, temporo-occipital

NS, not statistically significant

NR, not reported
